# The rates of premature chromosome condensation and embryo development after injection of irradiated sperms into hamster oocytes

**Published:** 2013-05

**Authors:** Sahar Moghbelinejad, Hossein Mozdarani, Zahra Rezaeian

**Affiliations:** 1 *Department of Medical Genetics, Faculty of Medical Sciences, Tarbiat Modares University, Tehran, Iran.*; 2 *Infertility Center, Shariati Hospital, Tehran, Iran.*

**Keywords:** *Ionizing radiation*, *Human sperm*, *Fertilization rate*, *Premature chromosome condensation*, *Hamster oocyte*

## Abstract

**Background: **Irradiation is one of the major causes of induced sperm DNA damage. Various studies suggested a relation between sperm DNA damage and fertilization rate after intra-cytoplasmic sperm injection (ICSI).

**Objective:** In this study, fertilization rate and premature chromosome condensation (PCC) formation after ICSI of hamster oocytes with irradiated sperms from normal and oligosperm individuals was investigated.

**Materials and Methods: **Human sperms were classified according to counts to normal and oligosperm. Ten samples were used for each group. Golden hamster oocytes were retrieved after super ovulation by PMSG and HCG injection. From retrieved oocytes, 468 were in metaphase II. Control and 4 Gy gamma irradiated sperms were then injected into oocytes. After pronuclei formation in injected oocytes and formation of 8 cells embryos, slides were prepared using Tarkowskie's standard air-drying technique. The frequency of embryos and PCC were analyzed using 1000× microscope after staining in 5% Giemsa.

**Results:** The extent of embryo development in oocytes injected by irradiated sperms was lower than those injected by non-irradiated sperms (p=0.0001). The frequency of PCC in failed fertilized oocytes was significantly higher in oligosperms (46%) compared with normal ones (0%), but there was no significant difference between irradiated and non-irradiated samples in each group (p=0.12).

**Conclusion:** The results showed that irradiation of sperms might influence the fertilization outcome possibly due to sperm DNA damage. One possible cause of precluding oocytes from fertilization in oligosperm individuals might be the formation of PCC.

## Introduction

The defect in genomic material may take the form of chromatin condensation abnormality, DNA breaks or DNA integrate defects that are known as sperm DNA damage. Sperm DNA damage is clearly associated with abnormal spermatogenesis and male infertility (1). One of the major factors resulting in defective sperm function is oxidative stress (OS). OS occurs when production of reactive oxygen species (ROS) by leukocytes or spermatozoa is excessive, and/or the antioxidant capacity of semen decreases (2). The variety of experiments has shown that irradiation is one of the exogenous sources of ROS production and DNA damage in sperm and causes temporary and permanent infertility (3-5).

Irradiation induces sperm aneuploidy, structural chromosome aberrations, chromatin structure anomalies, DNA breaks, and higher frequency of mutations. High levels of induced DNA damage in human sperm by *in vitro* or *in vivo* (radiotherapy in cancer patient) irradiation is also reported (6-10). The varieties of studies have shown the impact of sperm DNA damage on fertilization rate and outcomes after using assisted reproductive techniques (ART). 

However, some other studies have found lack of correlation between levels of DNA damage and fertilization rates, but rather an association between DNA damage and post fertilization embryo development has been noticed. This might be related to the degree of DNA damage (11, 12).

On the other hand, studies of failed fertilized human oocytes showed that after aneuploidy the next prevalent cause of failed fertilization in both IVF and ICSI is sperm premature chromosome condensation (PCC) (13, 14). In general, fusion of a mitotic cell like a mature oocyte and an interphase cell will induce PCC in the interphase nucleus. Following the entry of a sperm into the ooplasm, the oocyte becomes activated which results in completion of meiosis and formation of both male and female pronuclei. In such a condition, the oocyte remains at metaphase II while the sperm chromatin undergoes pre mature chromosomal condensation. Other than oocyte inactivation and oocyte cytoplasmic immaturity in induction of PCC, studies suggest that sperm genomic anomalies are contributing to this phenomenon too (13, 14).

There are many cases of people who are exposed to various doses of ionizing radiation for different reasons, such as living in high background radiation environment, job requirements and cancer treatment. A great number of these people might be candidates for ART methods. In this research we investigated possible relationship between radiation induced sperm DNA damage with fertilization rate and embryo development as well as PCC formation in failed fertilized oocytes after injection of irradiated sperm of oligozoospermic and normozospermc men into golden hamster oocyte. 

## Materials and methods

**Donors,** **sperm preparation and irradiation**

The sperm donors were divided into two groups according to the World Health Organization criteria (WHO 2010), i.e., oligozoospermic with mean sperm count of (15±2.4×10^6^), and fertile males with mean sperm count of (50±3.1×10^6^) (15). The ranges of age in these individuals were 23-48 and 22-47 years respectively. Each group consists of 10 donors that referred to the Fertility and Infertility Center, Shariati Hospital (Tehran, Iran). It should be noted that human sperm samples were used because the aim of this research was to study the impact of human sperm damage on fertilization rate among fertile and infertile patients. 

Normal samples were obtained from men referring to the infertility clinic because of infertility problems of their spouses, and they themselves had no indications of infertility problem. This experimental study was approved by the Ethical Committee of the Faculty of Medical Sciences of Tarbiat Modares University (Tehran, Iran). Patients gave their informed written consent. All samples had been screened to exclude radiation exposure, smoking, varicocele, genital tract infections, hepatitis, and HIV. In all cases, after 3 days of sexual abstinence, semen samples were collected by masturbation in sterile containers and were transferred to laboratory immediately after ejaculation. 

The semen samples were kept at 37^o^C for 30 min to complete liquefaction, and then the total sperm counts, number of sperm/ml, motility, and forward progression were determined according to WHO criteria. Sperms were processed by *Swim-Up* technique, and then they were irradiated to 4 Gy gamma-rays generated from a Co-60 source (Theratron II, 780C, AECL, Kanata, Canada) with a dose rate of 1.23 Gy/min.

**Hamster care and ovulation induction**

The Golden hamster were cared and used according to the guide for the care and use of laboratory animals of Tarbiat Modares University (Tehran, Iran) and housed under a 14 h light: 10 h dark regimen and 23^o^C temperature. A total of 40 adult female hamsters 5-7 weeks old were super ovulated using intraperitoneal injection (IP) of 25-30 IU pregnant mare serum gonadotropin (PMSG, Folligon; Intervent) followed with another injection of 25-30 IU human chorionic gonadotropin (hCG, Organon) hormone 48-56 h later. 

The cumulus cells mass contains eggs in the oviduct, 15-17 h later. Each hamster yield approximately 15-30 eggs. Hams-F10 medium (Biochrom.K.G) with some modification was used for sperm preparation, egg preparation and ICSI (Intra cytoplasm sperm injection) procedure. This media was supplemented with 15% fetal bovine serum (Sigma). Media was incubated in 5% CO_2_ at 37^o^C.

**Preparation of eggs**

The hamsters were sacrificed by cervical dislocation; the oviducts were excised through a dorsal incision through the skin and peritoneum which were closed later with sutures. The oocyte-cumulus complexes were recovered by rupturing the swollen ampulla portion of the oviducts in pre-warmed Hams-F10 37^o^C medium under mineral oil. The cumulus cells mass was transferred to a solution of 0.1% hyaloronidase (Sigma) to remove the cumulus corona cells from the oocyte (16, 17). The oocytes were thoroughly washed in the Hams-F10 medium several times and observed under inverted microscope (Nikon, Tokyo, Japan) at 200× magnification. Then, morphologically normal oocytes that had reached Metaphase II (MII) were selected for ICSI. 

**Intra cytoplasmic sperm injection procedure**

ICSI procedure was similar to the one which is used in infertility session of Shariati Hospital (Tehran, Iran). Oocytes were transferred to Hams-F10 medium with 15% fetal bovine serum (Sigma) under oil in 1006 Falcon Petri dishes. Sperm samples were introduced into a droplet of PVP (Sigma, USA). Eppendorff micromanipulator, mounted on Nikon inverted microscope (Nikon, Tokyo, Japan), was used to perform the sperm injection. Sperms with the best morphology were immobilized and sucked into an injection pipette. The sperms were injected into oocytes and then injected oocytes were incubate in incubator with CO_2_ 5% at 37^o^C.

**Checking of fertilization rate** **and slide preparation**

Injected oocytes reached pronuclei levels about 15h after ICSI. About 6-8 h after pronuclei formation, 4-8 cells embryos were formed. The number of failed fertilized oocytes, oocytes with pronuclei, and 2, 4, 8 cells embryos for each samples were then analyzed under inverted microscope (Nikon, Tokyo, Japan) at 200× magnification. 

To study frequency of PCC in failed fertilized oocytes, oocytes were fixed on slides. Failed fertilized oocytes and 2-8 cells embryos were fixed by Tarkowskie’s air drying technique with a slight modification (18). Eggs were treated with sodium citrate 1% (Sigma) as hypotonic solution for 15-20 min. Sequential fixation procedure was used for eggs in three stages as follows:

Fix I: Consisting of methanol (Merck), glacial acetic acid (Merck) and distilled water (5:1:4) for 3 min.

Fix II: Consisting of methanol, and glacial acetic acid (3:1) for 10 min

Fix III: Consisting of methanol, glacial acetic acid, and distilled water (3:1:1) for 60s.

Slides were stained in 5% Giemsa solution and were analyzed using 1000× light microscope. 

**Statistical analysis**

Statistical analyses including mean, standard deviation (SD), and non-parametric Mann Whitney U-tests were carried out using SPSS software (Ver. 16). P<0.05 was considered as statistically significant***.***

## Results

**Injection of unirradiated and irradiated sperm samples into hamster oocytes**

From 468 metaphase II oocytes after ICSI, the average frequency of embryos in fertile group was 37% and 30% (p=0.22) before and after irradiation respectively; this was 32% and 22% in oligospermic (p=0.11) samples. In spite of considerable difference in the rate of embryo formation in two groups, there was no statistical significant difference between them (Table I). Similar results were obtained for proneuclei formation in fertile and oligozoospermic samples before and after irradiation with no significant difference (p=0.13) (Table I).

**Progression of injected oocytes to 2, 4 and 8 cell embryos**

**Figure 1:**
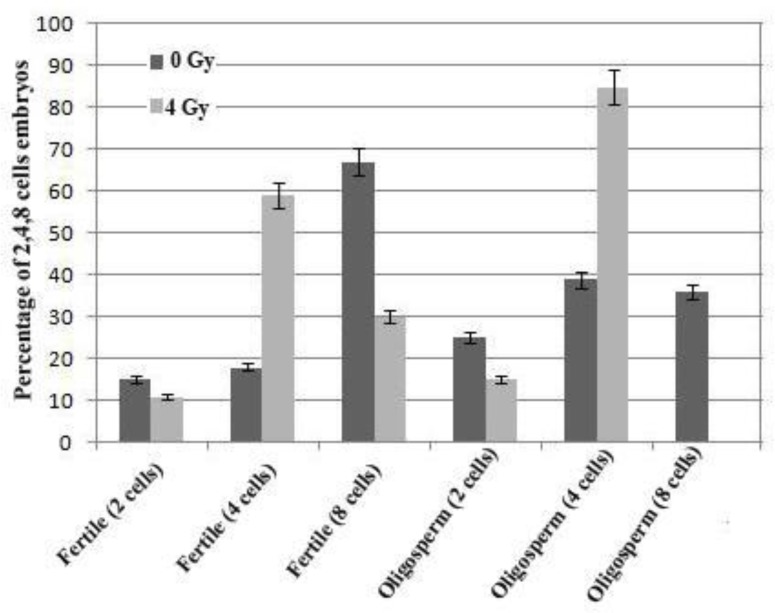
Frequency of 2, 4, 8 cell embryos in fertile and oligozoospermic samples before and after sperm radiation. Decreasing the frequency of 8 cells ebmryos in each group after sperm radiation is indicative of the extent of induced DNA damage in sperm

As shown in table I and figure 1, there was no significant difference between the frequency of two cell embryos before and after irradiation in fertile and oligospermic groups (p=0.33 and p=0.07 respectively). However, pre- and post-irradiation analysis of 4 cell embryos in fertile and oligospermic samples showed significant difference (p=0.001). 

The frequency of the 8 cell embryos decreased significantly from 67% to 30% after irradiation in fertile samples (p=0.001); this reveals that the majority of cells were blocked at 4 cell stage in these samples. No 8 cell embryos was formed in oligozoospermic samples after irradiation (p=0.001). 

**Frequency of sperm PCC in failed fertilized oocytes**

The rate of PCC formation after ICSI of un-irradiated sperms from fertile individuals was 0 compared with 3% in irradiated sperm samples (p=0.21). No statistical significant difference in the rate of PCC was found in unirradiated and irradiated sperm samples from oligozoospermic individuals (p=0.42). However, the rate of PCC in un-irradiated and irradiated samples of oligozoospermic patients was considerably higher than those of fertile individuals (p=0.001) )Table I).

**Table I:** Comparison of fertilization rate, embryos progression, PCC frequency in fertile and oligozoospermic samples before and after sperm irradiation

**Samples**	**Sperm treatment**	**No. of samples**	**No. of injected oocytes**	**Failed fertilized oocytes**	**Embryo frequency**	**Pronuclei**	**2 cell embryos**	**4 cell embryos**	**8 cell embryos**	**PCC**
Fertile										
	0 Gy	10	120	81 (67%)	45 (37%)	20 (24%)	7 (15%)	8 (18%)	30 (67%)	0 (0%)
	4 Gy	10	122	88 (72%)	37 (30%)	29 (33%)	4 (11%)	22 (59%)	11 (30%)	3 (3%)
Oligosperm										
	0 Gy	10	110	74 (67%)	36 (32%)	18 (24%)	9 (25%)	14 (39%)	13 (36%)	36 (49%)
	4 Gy	10	116	90 (77%)	26 (22%)	22 (24%)	4 (15%)	22 (85%)	0 (0%)	40 (44%)

P< 0.0001 compared to 4 Gy.   P< 0.0001 compared to oligoperm's groups (0 Gy and 4 Gy

## Discussion

Men are exposed to various doses of ionizing radiation because of their occupation, living in regions with high natural background radiation, and specially cancer treatment. Exposure of their germ cells to ionizing radiation can cause impaired spermatogenesis. Different studies about cancer patients showed that depending on the underlying disease, age, type, doses of radiation used, and duration of radiotherapy, these patients might present a post-therapy reproductive dysfunction as oligozoospermia and azoospermia, with 15-30% remaining sterile in the long term (19-22). 

Therefore, in this study we have investigated the fertilization rate and outcome of irradiated sperm with gamma radiation, after injection into golden hamster oocyte. We didn’t use human oocytes because of ethical issues, and on the other hand the variety of studies showed that human sperm would penetrate to hamster oocyte and form pronuclei and 2 cell embryos (23-25). In this study we showed that human-hamster embryos can progress to 8 cells. In radiotherapy of cancer patients cumulative radiation exposure to the testicles is about 3.56 Gy; therefore, we chose 4 Gy dose to study the effects of sperm irradiation on fertilization rate and outcome after ICSI.

Ionizing radiation is one of the exogenous sources of sperm DNA damage. Various studies have done regarding the relationship between sperm DNA damage and fertilization rate after ICSI. Most of them have shown a significant negative association between sperm DNA damage and fertilization rate after ICSI. Some studies have found no correlation between levels of DNA damage and fertilization rates. 

These findings suggest that ICSI bypasses the natural selection mechanisms and allows spermatozoa with DNA damage to fertilize oocytes, but repair system in oocytes involve in repairing sperm DNA damage (26, 27). On the other hand, our results showed no significant difference, in embryo formation before and after irradiation in studied groups (Table I) and confirmed other results which showed no association between sperm DNA damage and fertilization rate.

Different evidences show association between sperm DNA damage and post fertilization embryo development. In this regard some investigations indicate that embryos derived from spermatozoa with damaged DNA have a lower potential for later cleavage and reaching blastocyst stage. Some of the researchers believe that the implantation and pregnancy outcome would not be affected by using sperms carrying DNA damage because of embryos derived from damaged sperm DNA have little chance to reach blastocyst stage (28, 29). It is suggested that this process depends on the level of sperm DNA damage; as low level of DNA damage in sperm is insufficient to stimulate cell cycle arrest, apoptosis prior to implantation and may be expressed after pregnancy and caused pregnancy failure or effect in fetal or postnatal development (30, 31). 

In this research we showed that injected hamster oocytes with human sperm would not precede more than 8 cells; hence, fertilization outcome was followed up to 8 cells. In fertile groups with normal spermatogenesis most of the injected oocytes progressed to 8 cells before irradiation (67%), but after irradiation frequency of 8 cells embryos decreased significantly (30%). Activation of genomic expression in embryos occurs at 4-8 cell stage in human embryos, suggesting that the paternal genome may not be effective before 4-cells stage (32, 33). Our results indicated that in oligozoospermic samples no 8 cells embryos were formed in irradiated samples, and all of the embryos were blocked in 4 cells stage (85%). Our finding showed that all of the embryos stayed in 4 cells stage after induced DNA damage. Thus, ionizing radiation as damaging agent, which induced sperm DNA damage, can affect embryo development. 

Comparison of embryo formation between fertile and oligozoospermic samples showed that the frequency of embryo progression to 8 cells in oligozoospermic samples was lower than fertile ones, before and after irradiation Therefore, our observation was in line with the study which indicates that the nature and level of DNA damage differs between normal and infertile men (34). A dramatic distinctive difference between DNA damage in sperms of normal and different groups of subfertile individuals has recently been reported (35). The presence of PCC in failed fertilized oocytes indicates that successful penetration of sperm into the ooplasm of metaphase II oocyte is not a sufficient condition for achieving fertilization. However, PCC formation is affected by so many factors both for oocytes and sperm abnormalities (13, 14). In this study we used hamster oocytes to keep the ooplasmic condition similar for all samples. Our data showed no significant difference in PCC frequency before and after irradiation in each group. 

Different studies suggest that chromatin anomaly, especially protamine deficiency and impairment of chromatin packaging are the major reasons for PCC formation. Sperms with condensed nucleus, in G1 stage, when entering metaphase II oocytes, are protected from PCC formation because active mitosis promoting factor (MPF) does not react with protamine-associated DNA (36-39). On the other hand, Nili *et al* reported that sperm protamine concentration has a negative correlation with DNA fragmentation, indicating a possible protective role of protamine against sperm DNA damage (35).

Mozdarani *et al* in another study showed that the frequency of PCC in oligozoospermic and asthenozoospemic infertile men was higher than normal men. They concluded that this phenomenon might be due to chromatin abnormalities, improper DNA packaging, or chromosomal abnormalities in sperm of studied individuals (40). Our result is in agreement with these reports because the frequency of PCC in oligozoospermic samples was higher than normal ones, before and after irradiation (p=0.001).

## Conclusion

In conclusion, sperm damage due to radiation does not significantly influence fertilization rate, but it can decrease the embryo development. This decrease can be seen in oligozoospermic samples more than normal ones. The rate of PCC in oligozoospermic samples, as one of the factors of failed fertilization of oocytes, was more than normal ones.
